# The Potential Biological Roles of Circular RNAs in the Immune Systems of Insects to Pathogen Invasion

**DOI:** 10.3390/genes14040895

**Published:** 2023-04-12

**Authors:** Muhammad Nadeem Abbas, Saima Kausar, Isma Gul, Jisheng Li, Huijuan Yu, Mengyao Dong, Hongjuan Cui

**Affiliations:** 1 State Key Laboratory of Resource Insects, Southwest University, Chongqing 400716, China, ,; 2 Cancer Center, Medical Research Institute, Southwest University, Chongqing 400716, China; 3 Jinfeng Laboratory, Chongqing 401329, China

**Keywords:** noncoding RNA, circRNAs, insects, immune responses, host–pathogen interaction

## Abstract

Circular RNAs (circRNAs) are a newly discovered class of endogenously expressed non-coding RNAs (ncRNAs). They are highly stable, covalently closed molecules that frequently exhibit tissue-specific expression in eukaryotes. A small number of circRNAs are abundant and have been remarkably conserved throughout evolution. Numerous circRNAs are known to play important biological roles by acting as microRNAs (miRNAs) or protein inhibitors (‘sponges’), by regulating the function of proteins, or by being translated themselves. CircRNAs have distinct cellular functions due to structural and production differences from mRNAs. Recent advances highlight the importance of characterizing circRNAs and their targets in a variety of insect species in order to fully understand how they contribute to the immune responses of these insects. Here, we focus on the recent advances in our understanding of the biogenesis of circRNAs, regulation of their abundance, and biological roles, such as serving as templates for translation and in the regulation of signaling pathways. We also discuss the emerging roles of circRNAs in regulating immune responses to various microbial pathogens. Furthermore, we describe the functions of circRNAs encoded by microbial pathogens that play in their hosts.

## 1. Introduction

A large portion of the genome of living organisms is transcribed but not translated into proteins. For example, only 2% of RNA transcripts are translated into proteins in humans [1]. In recent years, it has been demonstrated that RNA transcripts that lack the ability to encode proteins play an important biological role in regulating various physiological processes. These transcripts are referred to as non-coding RNAs (ncRNAs), and they are divided into two major groups based on their functions, which are regulatory ncRNAs and structural ncRNAs [2]. Structural ncRNAs include transfer RNA (tRNA) and ribosomal RNA (rRNA). In both prokaryotic and eukaryotic cells, rRNAs are essential components of ribosomes, where they can be a physical component of both large and small subunits [3]. tRNAs consist of 76 to 90 nucleotides and are involved in the protein synthesis process by acting as a structural component along with mRNA and ribosomal RNA to form a polypeptide chain [4]. Regulatory ncRNAs are categorized into two main groups based on their size: small non-coding RNAs that are less than 200 nucleotides in length and long non-coding RNAs that are greater than 200 nucleotides in length [5,6]. Small regulatory RNAs have been identified in a variety of forms, with microRNAs (miRNAs), piwi interacting RNAs (piRNAs), and endogenous small-interfering RNAs (siRNAs) being the groups that have received the most attention from the researchers [7].

Circular RNAs are a large group of endogenous ncRNAs that are generated during a non-canonical splicing event called back-splicing. Back-splicing involves the formation of a covalent link between a downstream splice-donor site and an upstream splice-acceptor site. Despite the fact that they are not produced through a back-splicing mechanism, viroids were the first circRNA molecules to be discovered more than 40 years ago [8]. An electron microscopy analysis of cytoplasmic fractions of animal cell lines later revealed the presence of circRNAs in the cells [9]. At the time, only the testis-specific circRNA from the sex-determining region Y (Sry) gene was thought to have a function in mouse testis [10]. However, prior to this discovery, the vast majority of these circRNAs were thought to be ‘junk’ produced via aberrant splicing events [11]. Thousands of circRNAs in eukaryotes have been identified in recent years using high-throughput RNA sequencing (RNA-seq) and circRNA-specific bioinformatics algorithms. These circRNAs have been found in both invertebrates (e.g., fungi, protists, plants, worms, and insects) and vertebrates (e.g., fish and mammals) and have been demonstrated to have tissue-specific expression patterns [12,13,14,15,16,17,18].

A growing body of evidence, aided by cutting-edge sequencing and annotation technologies, has shed light on the biological role of circRNAs in a variety of physiological and pathological processes in living organisms [19,20,21,22]. CircRNAs play crucial functions in innate immune responses to a variety of pathogens [23]. Furthermore, circRNAs, which appear to regulate immune responses during microbial infection, are aberrantly expressed in invertebrates, including insects [24]. Despite the fact that the importance of circRNAs in invertebrates’ and vertebrates’ physiological functions is well established, research into their involvement in the immunological functions of insects is still in its infancy. Therefore, circRNAs research will not only improve our understanding of the molecular mechanisms underlying microbial infection, but will also provide future management strategies to control disease outbreaks among commercial insects as well as to control insect pests. In this review, we describe newly discovered circRNAs implicated in gene expression regulation during host–pathogen interactions. We also highlight future research perspectives.

## 2. Biosynthesis of CircRNAs

**Figure 1:**
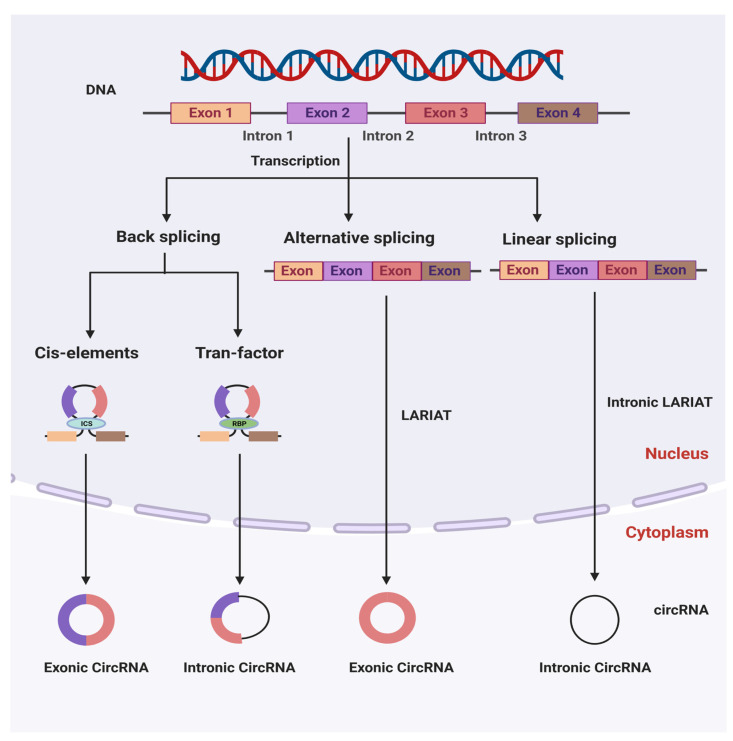
The molecular mechanisms of circular RNA biosynthesis and their classification in a cell. ICSs (intronic complementary sequences) and RBPs (RNA-binding proteins) modulate the production of circRNA.

Circular RNAs were discovered in RNA viruses for the first time by a group of researchers in 1976 [8]. CircRNAs have since been discovered in eukaryotic cells and yeast mitochondria [9,25]. As a result of recent advances in molecular techniques, including high-throughput sequencing technology and microarray techniques, researchers have recently discovered a diverse range of circRNAs in various organisms in nature. CircRNAs are typically produced through ‘back-splicing’ events that occur mainly after the synthesis of precursor messenger RNAs (pre-mRNAs), in which a downstream 5′ splice donor is linked to an upstream 3′ splice acceptor via a 3′→5′ phosphodiester bond [26,27]. CircRNAs are classified into three types based on their components: exonic circular RNAs [28], intronic circular RNAs [29], and exon–intron circular RNAs [30], with exonic circular RNAs accounting for the vast majority of the total. The biosynthesis of circRNAs is mainly dependent on cellular splicing machinery [31]. In contrast, in *Drosophila melanogaster* cells, inhibiting the spliceosome by depleting components of the U2 snRNP resulted in an increase in the ratio of circular to linear RNAs [32]. Therefore, it has been proposed that by slowing down pre-mRNA processing events, nascent RNA can be directed to alternative pathways that facilitate back-splicing [32]. A similar observation was demonstrated in another study on *D. melanogaster*, where splicing factor depletion was found to result in an increase in the level of circRNAs. Because additive effects were observed when multiple factors were depleted, it appears that each splicing factor may play an essential but non-redundant biological role in the formation of circRNAs [33]. A number of biogenetic pathways might be involved in the production of circRNA, including a complementary sequence-mediated circularization pathway, a lariat-driven circularization pathway, and an RNA-binding protein-mediated circularization pathway [34]. Some introns, such as Alu elements, which include both splice sites and flanking inverted complementary repeats, have been discovered to be required for the circularization of the intervening exons in cells. In addition, intronic complementary sequences (ICSs) and RNA-binding proteins (RBPs) have a role in circRNA generation regulation [35]. To facilitate back-splicing, the intronic repeat sequences must be base-paired with each other. Notably, when a pre-mRNA contains multiple intronic repeat sequences, the competitive pairing between the repeat sequences results in alternate circularization, which influences the splicing process [36]. A single gene, for example, may end up producing a variety of distinct circRNA transcripts as a result of this alternate circularization [37]. Exon skipping is another method of circRNA production in which a lariat precursor with one or more skipped exons is initially produced [38,39]. The lariat then removes its own internal intron sequences, resulting in the production of a mature circRNA and a double lariat. In certain circumstances, the intervening introns within the encircled exons are not removed, resulting in the generation of the exon–intron circular RNA [27]. Additionally, some RNA-binding proteins, such as the muscle blind, nuclear factor 90/nuclear factor 110 (NF90/NF110), and alternative splicing factor Quaking (QKI), have been found to increase back-splicing events by strengthening the interaction between upstream and downstream introns [40,41,42]. CircRNAs are generated by intron lariats that cannot be degraded or debranched, and they lack linear 3′ tails [29]. Despite the fact that the models described above provide some insight into the molecular mechanism of the circRNA generation, more research is needed to precisely understand the mechanisms underlying the process (Figure 1).

## 3. Functional Mechanism of CircRNAs

The circRNAs appear to be a diverse class of non-coding regulatory RNAs with a diverse range of functions, localization, and characteristics [43,44]. The biological roles of circRNAs are likely to be determined by their subcellular localization, i.e., in the nucleus or cytoplasm. The research shows that circRNAs can not only regulate the expression of genes in the nucleus, but they can also act as decoys for miRNAs and proteins, as well as create scaffolds for the formation of circRNA–protein complexes. There is evidence that certain circRNAs may act as templates for translation or as sources for the generation of pseudogenes [43,45,46]. Here, we describe the molecular functions of circRNAs and the roles they play in the nucleus and cytoplasm.

### 3.1. Functions of CircRNAs in the Nucleus

CircRNAs play a diverse biological role in the nucleus of a cell, including chromatin looping, transcription regulation, and alternative splicing [30,47,48,49]. For example, Conn et al. [47] discovered a circRNA called circSEP3 in the cell nucleus of Arabidopsis thaliana, which has been shown to be involved in modulating the splicing of SEPALLATA3, a homeotic MADS-box transcription factor important for floral homeotic phenotypes. In this particular example, circSEP3 is generated from exon 6 of SEP3, and it forms an RNA–DNA hybrid with its cognate DNA. This causes a pause in the transcription process, which is then followed by exon 6 skipping, resulting in an alternative splicing of SEP3 mRNA. In Zea mays, RNAs transcribed from centromeric retrotransposons were shown to have back-splicing; the resulting circRNAs bind to centromeres and promote chromatin looping by generating R-loops in these regions of the genome [49]. Therefore, it is important to investigate which molecular mechanisms are involved in the retention of centromeric circRNAs in the nucleus and how these circRNAs interact with the genome in order to regulate gene transcription at the nuclear level.

### 3.2. Functions of CircRNAs in the Cytoplasm

Following their generation, most circRNAs are transported from the nucleus to the cytoplasm. CircRNAs have been shown to play a variety of biological roles in the cytoplasm, such as acting as decoys for miRNA, or serving as protein scaffolds, or sequestering proteins. There are some circRNAs in the cytoplasm that serve as competing endogenous RNAs (ceRNAs), which are defined as miRNA sponges that bind miRNAs, inhibiting them from binding and suppressing their target mRNAs [50,51]. Despite concerns about the number of ceRNAs and miRNA binding sites needed to be present to achieve a measurable effect within a given cell, researchers believe that many abundant circRNAs can act as sponges for miRNA [52,53]. In the mouse, circSry is possibly involved in the development of the testis by sponging miRNAs since there are 16 target sites for miR-138 on circSry [11]. CircHIPK2 can act as a sponge for miR124-2HG in human cells and can regulate the activation of astrocytes during endoplasmic reticulum stress and autophagy [54]. On the other hand, a high expression of circHIPK3 either improves cell proliferation or governs insulin secretion by acting as a sponge for a variety of miRNAs [55,56]. There have also been reports that circRNAs frequently interact with different types of proteins during their life cycle [44]. The following examples can be used to illustrate how circRNAs function as protein scaffolds. The circFoxo3 gene is highly expressed in the cytoplasm of mouse cells and is associated with the progression of the cell cycle. This is accomplished by interacting with the cell cycle proteins cyclin-dependent kinase 2 (CDK2) and cyclin-dependent kinase inhibitor 1 (p21), resulting in the formation of the ternary complex circFoxo3–p21–CDK2 and the inhibition of the CDK2 function, which is normally required for the progression of the cell cycle [57]. It was proposed that circFoxo3 promotes cardiac-cell senescence by interacting in the cytoplasm with the proteins that are associated with senescence, such as E2F1 and ID-1, as well as proteins that are associated with stress, such as hypoxia-inducible factor 1α (HIF1α) and FAK, thereby preventing FAK from localizing to mitochondria or HIF1α from translocating to the nucleus in stressed cells [58]. In addition, it has been shown that circACC1 can act as a metabolic adapter in response to serum deprivation by forming a ternary complex with the AMPK β and γ regulatory subunits, promoting the enzymatic activity of the AMP-activated protein kinase (AMPK), which acts as an enzyme in the AMPK metabolic cycle [59]. Furthermore, exon–intron-comprising circRNAs play an important biological role in the nucleus, which includes their ability to promote the transcription of their parent genes by interacting with the U1 small nuclear ribonucleoproteins and with Pol II at the promoters of their parent genes [30].

Many circRNAs can also exhibit the ability to sponge up proteins, which is an indication of their ability to bind proteins. By promoting the generation of circMbl, increased expression of the multifunctional protein MBL leads to decreased production of the linear Mbl mRNA; as a result, circMbl sequesters MBL and prevents it from performing other neural functions [40]. CircANRIL, which is associated with atherosclerotic cardiovascular disease, suppresses ribosome biogenesis in vascular smooth muscle cells and in macrophages by binding to the essential 60S ribosome subunit assembly factor pescadillo homologue 1, resulting in atherosclerosis-related nucleolar stress and cell death [60,61]. Finally, circPABPN1 is largely localized to the cytoplasm and inhibits the binding of the RBP HuR (also known as ELAVL1) to its cognate linear PABPN1 mRNA, resulting in reduced mRNA translation [62].

## 4. CircRNAs in Insects

**Figure 2:**
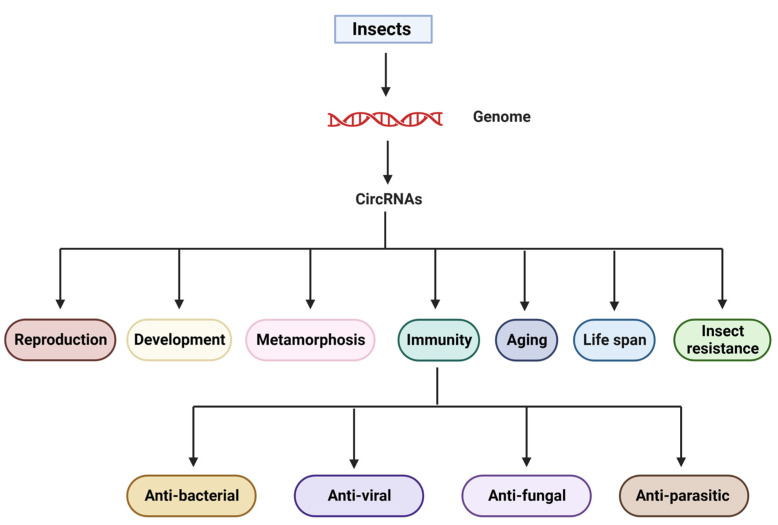
Overview of the biological role circular RNA plays in regulating various biological processes, in particular, immunity.

CircRNAs, as with most other vertebrates and invertebrates, have been demonstrated to exist in a wide range of insect species, particularly those whose entire genome has been sequenced. Researchers have been able to identify novel circRNAs by revising or discovering existing ones in recent years as the depth of sequencing platforms has increased. CircRNAs of insects are involved in various aspects of insect biology, including development [63], reproduction [64,65], metamorphosis [66,67], insecticide resistance, aging [68], life-span [69], and host–pathogen interactions and immunity [70,71], among others (Figure 2). These potential functions imply that they could be key regulators of insect physiological functions.

## 5. CircRNAs in the Innate Immunity of Insects

CircRNAs in insects have not been extensively studied so far, so the majority of their functions are still unclear. In insects, they may be produced in response to microbial pathogen infection to regulate immune responses. CircRNAs have been shown to perform biological functions in life processes in a variety of ways, such as miRNA sponges, protein sponges, translation proteins, biomarkers, and so forth [72]. Here, we describe examples of recent studies on circRNAs discovered in insects after microbial pathogen infection and their possible biological role in host–pathogen interactions (Table 1).

### 5.1. CircRNAs in Anti-Viral Immunity

Viruses, in general, are pathogenic agents that can replicate within host cells if the host cell provides the necessary resources. Because of this, viruses are considered to be obligatory intracellular pathogens with limited coding capacity. There are significant medical and economic costs associated with viruses that infect insects. So far, a large number of studies and review articles have been published that address the insect innate immunity and the molecules associated with immunity that is involved in the anti-viral processes of insects [73,74,75,76,77,78]. However, the biological role of circRNAs in the regulation of innate immunity during viral infection is still unknown.

*Bombyx mori* cytoplasmic polyhedrosis virus (BmCPV) is a silkworm-specific viral pathogen that can be transmitted via oral feeding [70,79,80]. The BmCPV specifically infects the epithelial cells of the silkworm midgut, and as the disease progresses, white wrinkles typically appear in the posterior part of the midgut. Consequently, the digestive and absorption functions of the midgut are severely impaired [70,71]. Silkworms utilize various defense mechanisms to inhibit or remove viral infections. A new class of ncRNAs (circRNAs) was recently discovered in insects and has been reported to play an important biological role in viral infection inhibition. Hu et al. [81] uncovered a novel biological function of circRNAs in anti-viral immune responses. The authors discovered thousands of circRNAs in BmCPV-infected and normal silkworm midguts, the vast majority of which were conserved. Based on the differential gene expression, they proposed that most of the circRNAs are up-regulated after BmCPV-infection in the midguts of silkworms. Moreover, the silkworm immune system generates immunoglobulin-like proteins and anti-viral factors. Interestingly, many of the immune genes that are up-regulated are the target genes of the downstream miRNAs of the up-regulated circRNAs [81]. Furthermore, Bmo-miR-278-3p has been shown to bind circRNA_9444, circRNA_8115, circRNA_4553, and circRNA_6649, and the miR-278-3p has the ability to negatively regulate the expression of the insulin-related peptide-binding protein 2 gene in silkworm larvae, while positively regulating the mRNA transcript levels of BmCPV [82]. These results suggest that miR278-3p may play an important role in BmCPV replication by interacting with host-genome-encoded circRNAs [81]. These results indicate that circRNAs may regulate the progression of BmCPV infection by binding miRNAs that control the expression of associated genes, though the precise regulatory mechanism remains unknown.

*B. mori* nucleopolyhedrovirus (BmNPV) causes diseases that have always been difficult to control, resulting in tremendous economic losses in the sericulture industry [83,84]. It has been proposed that BmNPV proliferation within host cells is mediated by a cascade of viral gene expression, and identifying host genes that respond to BmNPV infection is a promising strategy for developing BmNPV-resistant silkworms [85,86]. Despite this, several recent studies have investigated the effect of circRNA involvement in BmNPV infection [81,84,87]. It has been demonstrated that after BmNPV infection, the expression levels of circRNAs in the silkworm midgut are altered, and these dysregulated circRNAs may play roles in BmNPV infection, implying that these altered circRNAs may be a novel type of anti-viral factor [88]. In addition, Hu et al. characterized the altered circRNA and demonstrated that these circRNAs are linked to various biological functions and can modulate various immune pathways, such as the Notch signaling pathway, ABC transporters, and the endocytosis pathway. This study established circRNA–miRNA interaction networks and predicted the relationships between the circRNAs and miRNAs. The identified circRNAs were found to have a variable number of binding sites for miRNAs; for example, bmo-miR-3262 can bind to 34 circRNAs, and bmo-miR-3373-3p and bmo-miR-745-3p can interact with 30 and 29 circRNAs, respectively. CircRNA_0930, circRNA_2873, circRNA_5070, circRNA_0413, and circRNA_4553 contain multiple binding sites for miRNA, implying that circRNAs might regulate the progression of BmNPV infection by sponging to miRNAs that could regulate gene expression; however, further experimental confirmation is required to prove these initial outcomes. Another study also found the differential expression of circRNAs in the fat body of silkworms, along with differentially expressed miRNAs and 730 differentially expressed mRNAs that are associated with BmNPV infection. The circRNA/miRNA/mRNA analysis suggested that various immune signaling pathways (e.g., Wnt signaling pathway, phagosome, Hedgehog signaling pathway) are influenced that are possibly involved in impairing viral infection [87]. The expression of circ_0001432 in various tissues and at different infected time-points was widely detected in all tissues, and it could be up-regulated 24–72 h post-infection, suggesting that BmNPV infection triggered the immune response to be accomplished by circ_0001432 at the transcription level. Further experiments, such as the luciferase reporter assay for the validation of the interaction between circ_0001432 and its target miRNAs and knockdown or overexpression of circ_0001432 in vivo and in vitro, are needed to illustrate the function of circ_0001432 [87]. Furthermore, Zhang et al. [89] discovered 444 novel circRNAs from the BmN cells. Of these 75 circRNAs, some have been shown to be differentially expressed during BmNPV infection. Interestingly, the abundance of circRNAs has been reported to be higher in the control group than the BmNPV infection group, and only 1 of 75 differentially expressed circRNAs was up-regulated in the infection group, suggesting that BmNPV infection can widely induce the down-regulation of circRNAs in BmN cells. However, the mechanism remains to be explored why BmNPV infection down-regulates circRNAs in BmN cells. In contrast, various in vivo studies have proposed the up-regulation of circRNAs after BmNPV infection in silkworm [87,88]. The circRNA–miRNA–mRNA network indicates that the target genes modulate the metabolic pathway and immune-related signaling pathways, such as pantothenate and CoA biosynthesis, β-alanine metabolism, glyoxylate, and dicarboxylate metabolism, and citrate cycle [89]. Thus, it seems that circRNAs mainly control the metabolic processes in these cells. It is reported that the reproduction of BmNPV is highly dependent on host energy metabolism [90,91,92], suggesting that alteration in the metabolic activities of BmN cells influences viral infection. 

Rice black-streaked dwarf virus (RBSDV) is a species of the genus Fijivirus in the family Reoviridae. RBSDV is transmitted persistently by *Laodelphax striatellus* (Fallén) and causes severe losses in East Asian countries. A recent study determined the circRNA expression profile in *L. striatellus* midgut tissues that were either RBSDV free or RBSDV infected. After RBSDV infection, circRNAs were found to be differentially expressed, with a variable number of binding sites for miRNAs thought to control immune-pathway associate genes. A single miRNA was predicted to interact with multiple circRNAs, such as miR-14-3p, which could be bound by four differentially expressed circRNAs [89]. In addition, Zhang et al. [89] reported that circRNA2030 is up-regulated in the midgut of *L. striatellus* after RBSDV infection. Loss of functional analysis experiments has shown that the suppression of circRNA2030 can regulate the expression patterns of the parental gene phospholipid-transporting ATPase (PTA) and enhance RBSDV infection in *L. striatellus*, implying that circRNA2030 has antivirus functions in *L. striatellus* under RBSDV infection. Interestingly, this study also showed that the six miRNAs predicted to interact with circRNA2030 were also shown to be down-regulated after circRNA2030 was depleted. The possible molecular mechanism of circRNA2030 in regulating RBSDV infection might be via regulating the expression of PTA at the mRNA level; however, more research is required to fully understand the molecular mechanism [89].

Overall, even though numerous circRNAs from different species of insects have been identified thus far, this suggests that circRNAs play a vital biological role in anti-viral immunity in insects. However, how host receptors detect viral pathogens and which receptor types are crucial for anti-viral immunity are still unknown. It has been established that viruses activate Toll and IMD pathways, but it is unclear how viruses do so and whether PRRs or other sensors are involved. It is interesting to note that only Dicer-2, a receptor for viral double-stranded (ds) RNA, has been identified in insects. Dicer-2 has been suggested to have a dual biological role in anti-viral immunity, activating both anti-viral RNAi and signaling that results in the expression of anti-viral molecules such as Vago [93,94,95]. In vertebrates, PRRs belong to different structural families, such as Toll-like receptors, melanoma differentiation-associated gene 5, RIG-I-like receptors, nucleotide-binding domains, and leucine-rich repeat-containing receptors. These proteins interact with viral RNAs to generate anti-viral effector molecules, which consequently lead to the elicitation of anti-viral immune responses [96,97,98,99]. Recently, Chen et al. [100] reported on a potential molecular mechanism, revealing a novel role for circRNAs in inducing anti-viral immune responses. They discovered that the delivery of purified circRNAs activates RIG-I and provides a strong immune defense against viral infection. Further analysis suggested that self-splicing intron-derived circRNAs, rather than the same circRNAs produced with endogenous introns and spliced by cellular splicing machinery, are the ones that activate RIG-I; consequently, the splicing mechanism of circRNAs is necessary for the activation of immune responses. In host cells, a set of diverse RNA-binding proteins bind to the endogenous cellular circRNAs. In contrast, ‘non-self’ circRNAs rarely interact with proteins. The RNA-binding proteins are used to identify ‘self’ circRNAs and help host cells to distinguish them apart from foreign circRNAs. 

### 5.2. CircRNAs in Anti-Bacterial Immunity

**Figure 3:**
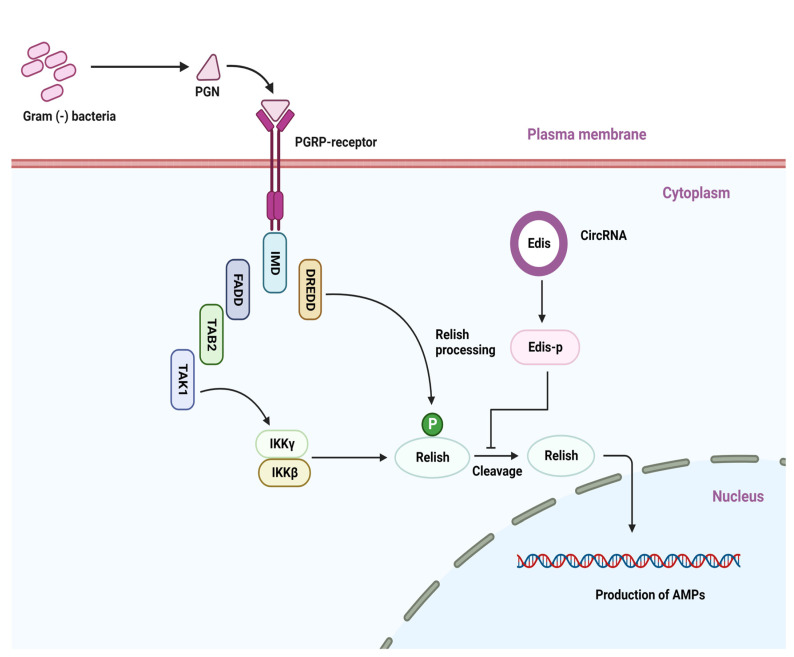
Representative circular RNAs play a biological role in the modulation of the IMD signaling pathway. The PGRP receptor recognizes various bacterial components and induces a downstream signaling cascade.

Both the IMD (activated against Gram-negative bacteria) and Toll (activated against Gram-positive bacteria) pathways have been shown to be involved in the inhibition of bacterial infection in insects and other invertebrates [49,83,101,102,103]. CircRNAs have recently been reported to be implicated in the regulation of anti-bacterial immunity [104]. Using *D. melanogaster* as a model organism, Xiong et al. [104] confirmed the involvement of circRNAs in anti-bacterial immunity. They used a combination of the molting hormone, 20-hydroxyecdysone, and a mixture of *Escherichia coli* and *Micrococcus luteus* to detect the anti-bacterial circRNAs, and they treated *Drosophila* S2 cells with bacteria, which activates innate immunity signaling [73,103]. Following the bacterial treatment described above, a total of ~5000 circRNA candidates were identified from *Drosophila,* and the majority of the identified circRNAs were found to be conserved across animal species [15,104,105]. Following this, a conserved circ_1705 (circ_Ect4) was isolated and named Edis (Ect4-derived immune suppressor), which is derived from a single exon of the Ect4 transcripts, and was proposed to regulate innate immunity [15,104,105]. Ect4 and Edis, respectively, promote and suppress optimal antimicrobial peptide gene expression in terms of immune regulation in neurons. Ect4 dampens the IMD anti-bacterial signaling in the tracheal epithelium but not in the fat body or digestive tract [104,106], implying that both circRNAs are actively involved in immune responses against bacteria. Edis may also suppress IMD innate immune signaling. In addition, Edis can code for a protein with a biological function. However, because the ORF of Edis terminates after only 1 nucleotide downstream of the back-spliced exon junction, it does not produce any distinctive amino acid sequences that could distinguish Edis-p from Ect4. The ability of endogenous Edis to generate Edis-p is still an unknown fact. Experimental evidence indicates that the neurodevelopmental phenotypes and the innate immunity hyperactivation triggered through Edis depletion are rescued by the ectopic expression of Edis-p, which not only phenocopies Edis overexpression. Additionally, Edis-p co-immunoprecipitated analysis suggested that Edis-p can inhibit endogenous Relish processing both in cells and in vitro. The simplest interpretation of this phenomenon is that the immune suppressor function of Edis is dependent, at least in part, on Edis-p [104]. Overall, Edis appears to be a novel circRNAs that has the ability to produce functional proteins that modulate immune responses [107,108]. Furthermore, Xiong et al. [104] found that in both the presence and absence of Gram-positive (*M. luteus*) bacterial infection, Edis depletion can affect the anti-bacterial IMD signaling pathway as well as up-regulate the anti-fungal peptide gene Drosomycin. Thus, Edis appears to have an effect on both branches of innate humoral immunity in *Drosophila* (Figure 3).

### 5.3. CircRNAs in Anti-Fungal and Anti-Parasite Immunity

Entomopathogenic fungi recognize and infect insects by adhering their spores to the cuticles of insects and forming special appressoria that allow the fungi to penetrate the cuticles. When fungal filaments enter into a body cavity or hemocoel of an insect, they transform into yeast-like cells that undergo budding in order to propagate rapidly and counteract the immune response of the hosts. When the infection cycle is complete, dead insects either mycose to produce asexual conidial spores or are colonized to form a fruiting body, which in turn produces sexual spores for the next infection cycle [109]. Entomopathogenic fungi are widely used to control agricultural pests but are also responsible for causing diseases in economically important insects [110,111,112,113]. Therefore, the resistance mechanism of insects is crucial to understand the precise management of fungal infection. In this section, we describe the biological role of circRNAs in the inhibition of fungal infection.

Honey bees not only pollinate a large number of wildflowers and crops, but they also produce a variety of API products, such as honey, royal jelly, propolis, bee pollen, and beeswax, thus playing critical ecological and economic roles. Honey bees, as eusocial insects, are susceptible to bacterial, fungal, and viral infections. The western honey bee (*Apis mellifera*), a eusocial insect with superior economic and ecological value, is widely used in global beekeeping [114]. CircRNAs were found to be altered in the *Apis mellifera ligustica* larval guts during the *Ascosphaera apis* infection and have been linked to host immune responses [115]. It has also been proposed that exonic circRNAs are the most abundant during fungal infection. Furthermore, it was reported that circRNAs greatly fluctuate during the *A. apis* infection in comparison to uninfected groups. These circRNAs in the larval guts are likely to play critical biological roles during an *A. apis* infection, as evidenced by their involvement in a variety of immune pathways, such as apoptosis, autophagy, endocytosis, as well as MAPK, Toll, and IMD signaling pathways. This indicated that the circRNAs likely perform extensive regulatory functions via modulating the transcription of source genes, though the impact may not be strong. In addition, three source genes encoding storage proteins (HEX110, HEX70B, and HEX70C) have also been suggested to be targeted by these circRNAs in larval guts infected by *A. apis*, implying that these circRNAs could affect storage protein synthesis by regulating the transcription of the related source genes and modulate the host immune defenses against an *A. apis* invasion [115]. These storage proteins found in holometabolous insects, including honey bees, not only provide the material basis for larval and pupal development but also play essential roles in various processes such as immunity and oviposition. Vieira et al. [116] found that miR-34 and miR-210 negatively regulated the expression of both HEX70B and HEX110 by directly and redundantly binding to their 3′ untranslated region (UTR) sequences. However, establishing the interaction between circRNAs–miRNAs-source genes is still an open question for the honey bee during fungal infection that requires further research. On the whole, circRNA-regulated source genes may regulate various cellular and humoral immune pathways, including apoptosis, autophagy, endocytosis, MAPK, Toll, and IMD signaling pathways. Another recent study challenged *Apis cerana cerana*, which is a subspecies of the eastern honeybee, *Apis cerana*, with *A. apis*. This study suggests that fungal pathogens have a strong impact on circRNAs, which are produced to counter infection, as a large number of circRNAs are modulated following the fungal infection; however, this study did not further explore the functions of circRNAs [44].

*Nosema ceranae* is a common unicellular fungal parasite that can infect honey bees and cause bee nosemosis. Infection with this parasite (*N. ceranae*) has been shown to influence circRNAs in the midgut of the honey bee *A. cerana*. In particular, *N. ceranae* invasion regulates circRNA production, and it appears that infection time has a significant impact on circRNA production. A recent study showed that the abundance of circRNAs varies with infection time, suggesting that the infection period is important for the induction of the host immune response. Interestingly, only a small number of circRNAs were found to be differentially expressed at different infection times in *A. cerana* after *N. ceranae* infection. These circRNAs modulate various immune pathways, including endocytosis, lysosomes, phagosomes, ubiquitin-mediated proteolysis, the metabolism of xenobiotics by cytochrome P450, and insect hormone biosynthesis [117]. In addition, evidence demonstrates that circRNAs have a crucial role in detecting damage caused to the midgut epithelial cell structure by *N. ceranae* infection. It also appears that the circRNAs of the host are employed to regulate source gene transcription, thereby modulating the Hippo signaling pathway to facilitate cell renewal and regulate the immune response [118,119]. Thus, it is evident that *N. ceranae* altered the expression pattern of circRNAs in the midguts of *A. c. cerana*, and the circRNAs are likely to regulate host cellular and humoral immune response to microsporidian infection [117]. Another study determined the impact of the parasite *Vairimorpha ceranae* on adult honey bees, which can cause diseases such as nosemosis. The infection caused by *V. ceranae* has been demonstrated to modulate circRNAs. Furthermore, these circRNAs were proposed to be involved in various immune-associated processes such as cellular renewal, cellular structure, carbohydrate and energy metabolism, and cellular and humoral immunity [80].

In summary, besides viral and bacterial pathogens, fungal and parasitic pathogens have also influenced the production of circRNAs. This fact has been established by various studies that were conducted by various research groups. Fungal or parasitic infections induce a plethora of circRNAs that are proposed to be involved in anti-fungal immunity. However, the molecular mechanism has not been established, but this knowledge will help researchers around the globe design studies to deepen our knowledge regarding anti-fungal immunity in insects. 

**Table 1:** Circular RNAs involved in the immune responses of insects to microbial pathogens.

CircRNA	Insect Species	Pathogen	Expression	Target	Effect	Reference
circRNA_9444, circRNA_8115, circRNA_4553 and circRNA_6649	*B. mori*	BmCPV	Up ↑	Bmo-miR-278-3p	Likely negatively regulate the insulin-related peptide-binding protein 2 expression while positively controlling the transcript levels of BmCPV	[82]
circ_0001432	*B. mori*	BmNPV	Up ↑	?	?	[87]
circRNA2030	*L. striatellus*	RBSDV	Up ↑	Likely target miR-14-3p, miR-9a-3p, miR-92a, and miR-315-5p	Regulates the expression patterns of phospholipid-transporting ATPase (PTA) and enhance RBSDV infection	[89]
Ect4	*Drosophila*	Escherichia coli and Micrococcus luteus	Up ↑	?	Promotes optimal antimicrobial peptide expression via the IMD pathway	[104,105]
Edis	*Drosophila*	*E. coli* and *M. luteus*	Up ↑	?	Suppresses antimicrobial peptide expression via repressing the IMD pathway	[104,105]

↑ upward arrow indicates the expression circRNA induced. ? question mark showed that relevant information is still undiscovered.

## 6. Pathogen-Encoded CircRNAs Hijack the Host System for Proliferation

At present, many different viral strains with various genome types, such as single-stranded DNA, double-stranded dsDNA, single-stranded RNA, and double-stranded RNA, have been described to encode circRNAs, and some of them have been demonstrated to be involved in biological processes. Despite the fact that viral circRNAs can be found in both the DNA viruses and RNA viruses, it has been observed that a large number of viral circRNAs have been identified and validated in the DNA viruses, such as human papillomaviruses (HPVs) and Hepatitis B virus (HBV) [120,121]. Some recent studies have confirmed that circRNAs are encoded by RNA viruses such as the *B. mori* cytoplasmic polyhedrosis virus (BmCPV) [122,123]. The viral circRNAs have a number of biological roles, including controlling viral over-proliferation and preventing innate immunity from being activated [124]. In this section, we highlight the effect of circRNAs that are encoded by pathogens on the immune systems of insects. 

*B. mori* nucleopolyhedrovirus (BmNPV) is one of the dangerous pathogens that infects the silkworm *B. mori*, and causes enormous economic losses to the sericulture industry. BmNPV contains over 140 protein-coding genes in its 128.4 kilobases pair-long double-stranded genome as well as generates circRNAs [73,125,126]. This viral pathogen has also been reported to produce a variety of circRNAs in order to evade host immunity. Recently, a viral circRNA-00010 was identified in BmN cells infected with BmNPV, and its expression level increased along with viral infection. This suggests that viral circRNA-00010 is the result of alternative splicing of a delayed early gene, the mechanism of which is known during viral infection. Interestingly, the level of expression of the viral replication markers (e.g., Bm59 gene and GFP protein) is increased in a dose-dependent manner in relation to the expression level of circRNA-000010, indicating that circRNA-000010 may play a pivotal role in viral replication in host cells [126]. The viral circRNA-00010 is localized in the cytoplasm and may act as mRNA to direct protein synthesis [127]. A significant proportion of circRNAs contained ORF that can be translated cap independently, such as internal ribosome entry site and N6-methyladenosine [128]. These translation elements were also discovered in viral circRNA-00010. Thus, it appears that viral circRNA-00010 exhibits anti-viral activity, likely through its translated product VSP39. In addition, circRNA-000010 can also sponge a number of silkworm miRNAs, such as bmo-miR-2808c, bmo-miR-2808a-3p, and bmo-miR-2739, suggesting that this circRNA may have multiple roles in the host cells [126]. Further studies that focus on the molecular mechanism of circRNA-000010 in the replication process of virus will further improve our understanding of viruses. 

CircRNAs have been identified in other pathogenic interactions, and functions for these circRNAs have been proposed; further experimental evidence is needed to understand their precise functions. For example, circRNAs from *A. apis*, a fungus that infects honeybee larvae, were recently discovered. These circRNAs appear to regulate gene expression by competitively binding miRNAs, and they have been proposed as potential biomarkers for chalkbrood [129]. Overall, there is very little knowledge regarding circRNAs encoded by pathogens, and cutting-edge new molecular techniques are hoped to accelerate research on circRNAs in pathogens. 

## 7. Host Hijack Pathogen Encoded CircRNAs to Inhibit Infection

The host immune system also has the ability to hijack circRNAs encoded by pathogens in order to inhibit the infection of that pathogen. In a study published recently, vSP27, a novel viral small peptide derived from the negative strand of S5 of BmCPV, was identified and shown to negatively regulate BmCPV infection [130]. The vSP27 peptide is translated by BmCPV from circRNA-vSP27, and it is considered that vSP27 could act as a catalyst for the induction of ROS inside cells, and interact with Akirin to activate the NF-κB signaling pathway against viral infection [122,130]. The relationship between ROS generation induced by vSP27 and BmCPV infection has recently been established. The vSP27 can induce ROS generation in BmN cells and can inhibit BmCPV infection. There is some evidence that N-Acetyl-L-cysteine, an antioxidant, reduces ROS levels in cells and increases viral infection. Furthermore, it was found that treatment with vSP27b resulted in the deterioration of mitochondrial functions and decreased activity of antioxidant enzymes in BmN cells. Thus, it appears that vSP27 induces mitochondrial damage, inhibits the activity of antioxidant enzymes, and promotes ROS production; thus, vSP27 inhibits BmCPV infection via ROS-dependent signal transduction. NF-κB is a transcription factor that becomes active in response to cellular stress, such as oxidative stress and viral infection [131,132]. The vSP27 can induce NF-κB, which in turn activates the NF-κB signaling pathway in BmN cells, leading to an increase in the production of antimicrobial peptides (e.g., CecB, CecA, and lysozymes). These antimicrobial peptides have also been reported to be induced by the co-expression of STING and Relish, suggesting that these antimicrobial peptides are induced through the activation of anti-viral immune responses by Relish [132]. Therefore, it seems that vSP27 activates the canonical NF-κB signaling pathway to produce antimicrobial peptides to inhibit BmCPV infection, although the anti-viral mechanism differs from the immune defense of the Toll and IMD pathways. In addition, Zhang et al. [122] investigated the molecular mechanism of vSP27 for the activation of the NF-κB signaling pathway using different molecular techniques. The authors suggested that vSP27 can directly interact with Akirin in the process of virus infection. Akirin regulates the NF-κB-mediated signaling pathway via binding to NF-κB’s promoter region of NF-κB. Furthermore, Akirin could directly positively regulate the NF-κB signaling pathway [122]. Overall, the anti-viral mechanism of vSP27 from the BmCPV-derived circRNA-vSP27 was played by the ROS or Akirin-dependent activation of the NF-κB signaling pathway.

Furthermore, another study conducted by the same research group has identified a viral circRNA_000048 whose sequence corresponds with that of the region 164–1245 nucleotides on the BmCPV genomic dsRNA S5 segment (GQ294468.1). The viral circRNA_000048 has been shown to be translated into a micropeptide, vsp21, with 21 amino acid residues in an IRES-dependent manner [123]. The expression of vcircRNA_000048 and vsp21 is increased with the progress of virus infection, suggesting that vcircRNA_000048 is associated with disease pathogenesis. The vsp21 encoded by vcircRNA 000048 attenuates viral replication as S5-sORF (vSP27). In the silkworm *B. mori*, cytoplasmic polyhedrosis resulting from BmCPV infection is a chronic disease that progresses over a period of 8 to 12 days. Thus, it has been proposed that the vsp21 encoded by vcircRNA_000048 can forestall the premature death of silkworms caused by rapid virus proliferation [123]. There is a possibility that the inhibitory activity of vsp21 on BmCPV replication may be similar to that of the egt gene of the baculovirus, which encodes an enzyme called ecdysteroid UDP-glucosyltransferase. There has been evidence that the expression of egt in silkworms after BmNPV infection can block the molting of infected larvae for a longer period of time, thus enhancing virus yield [123,133,134].

## 8. Conclusions and Future Perspectives

Over the last decade, we have gained a deeper understanding of the biological role of ncRNAs in the context of host–pathogen interactions through the use of transcriptomics analysis [24]. The study of ncRNAs has been a subject of a great deal of research in order to gain a better understanding as to how the defense system of the host responds to infection by the immunoproteome [24,71,80]. In recent years, there has been a growing body of evidence demonstrating that circRNAs are involved in immune regulation in living organisms. We described a number of aspects of circRNAs in this review, such as their classification, biological roles, molecular mechanisms of actions, and their possible functions in the interaction between host and pathogen. Using examples from the biological context, we provided insight into the functions that circRNAs play as miRNA sponges, scaffolds, and decoys. In spite of this, many circRNAs have yet to be determined in terms of their biological roles and/or their functional roles within various cellular processes. What we know about circRNAs leads us to believe that the responses of host circRNAs are of biological significance. Thus, there is a need for extensive research into the precise biological roles of circRNAs in host–pathogen interactions, particularly with regard to the manner in which pathogens regulate host circRNAs to improve their survival. There have been a number of new molecular biology techniques developed that are likely to improve our understanding of the biogenesis and biological roles of circRNAs. It is possible to use these techniques to understand the molecular mechanism by which circRNAs interact with other types of RNA and proteins at the pathogen and host levels. Due to this, it is suggested that future studies on the molecular basis of host responses to pathogenic/parasitic interactions should focus on circRNAs in order to provide new insights into the control of infections caused by pathogenic organisms.

## Data Availability

Not applicable.

## References

[1] Djebali S., Davis C.A., Merkel A., Dobin A., Lassmann T., Mortazavi A., Tanzer A., Lagarde J., Lin W., Schlesinger F. (2012). Landscape of transcription in human cells. Nature.

[2] Jarroux J., Morillon A., Pinskaya M. (2017). History, Discovery, and Classification of lncRNAs. Long Non Coding RNA Biology.

[3] Dahariya S., Paddibhatla I., Kumar S., Raghuwanshi S., Pallepati A., Gutti R.K. (2019). Long non-coding RNA: Classification, biogenesis and functions in blood cells. Mol. Immunol..

[4] Fiannaca A., La Rosa M., La Paglia L., Rizzo R., Urso A. (2017). nRC: Non-coding RNA Classifier based on structural features. BioData Min..

[5] Esteller M. (2011). Non-coding RNAs in human disease. Nat. Rev. Genet..

[6] Quinn J.J., Chang H.Y. (2016). Unique features of long non-coding RNA biogenesis and function. Nat. Rev. Genet..

[7] Naqvi A.R., Islam M.N., Choudhury N.R., Haq Q.M. (2009). The fascinating world of RNA interference. Int. J. Biol. Sci..

[8] Sanger H.L., Klotz G., Riesner D., Gross H.J., Kleinschmidt A.K. (1976). Viroids are single-stranded covalently closed circular RNA molecules existing as highly base-paired rod-like structures. Proc. Natl. Acad. Sci. USA.

[9] Hsu M.T., Coca-Prados M. (1979). Electron microscopic evidence for the circular form of RNA in the cytoplasm of eukaryotic cells. Nature.

[10] Cocquerelle C., Mascrez B., Hétuin D., Bailleul B. (1993). Mis-splicing yields circular RNA molecules. FASEB J. Off. Publ. Fed. Am. Soc. Exp. Biol..

[11] Capel B., Swain A., Nicolis S., Hacker A., Walter M., Koopman P., Goodfellow P., Lovell-Badge R. (1993). Circular transcripts of the testis-determining gene Sry in adult mouse testis. Cell.

[12] Wang P.L., Bao Y., Yee M.C., Barrett S.P., Hogan G.J., Olsen M.N., Dinneny J.R., Brown P.O., Salzman J. (2014). Circular RNA is expressed across the eukaryotic tree of life. PLoS ONE.

[13] Ivanov A., Memczak S., Wyler E., Torti F., Porath H.T., Orejuela M.R., Piechotta M., Levanon E.Y., Landthaler M., Dieterich C. (2015). Analysis of intron sequences reveals hallmarks of circular RNA biogenesis in animals. Cell Rep..

[14] Jeck W.R., Sorrentino J.A., Wang K., Slevin M.K., Burd C.E., Liu J., Marzluff W.F., Sharpless N.E. (2013). Circular RNAs are abundant, conserved, and associated with ALU repeats. RNA.

[15] Westholm J.O., Miura P., Olson S., Shenker S., Joseph B., Sanfilippo P., Celniker S.E., Graveley B.R., Lai E.C. (2014). Genome-wide analysis of drosophila circular RNAs reveals their structural and sequence properties and age-dependent neural accumulation. Cell Rep..

[16] Salzman J., Chen R.E., Olsen M.N., Wang P.L., Brown P.O. (2013). Cell-type specific features of circular RNA expression. PLoS Genet..

[17] Maass P.G., Glažar P., Memczak S., Dittmar G., Hollfinger I., Schreyer L., Sauer A.V., Toka O., Aiuti A., Luft F.C. (2017). A map of human circular RNAs in clinically relevant tissues. J. Mol. Med..

[18] Xia S., Feng J., Lei L., Hu J., Xia L., Wang J., Xiang Y., Liu L., Zhong S., Han L. (2017). Comprehensive characterization of tissue-specific circular RNAs in the human and mouse genomes. Brief. Bioinform..

[19] Guarnerio J., Bezzi M., Jeong J.C., Paffenholz S.V., Berry K., Naldini M.M., Lo-Coco F., Tay Y., Beck A.H., Pandolfi P.P. (2016). Oncogenic Role of Fusion-circRNAs Derived from Cancer-Associated Chromosomal Translocations. Cell.

[20] Wang K., Sun Y., Tao W., Fei X., Chang C. (2017). Androgen receptor (AR) promotes clear cell renal cell carcinoma (ccRCC) migration and invasion via altering the circHIAT1/miR-195-5p/29a-3p/29c-3p/CDC42 signals. Cancer Lett..

[21] Mehta S.L., Pandi G., Vemuganti R. (2017). Circular RNA Expression Profiles Alter Significantly in Mouse Brain After Transient Focal Ischemia. Stroke.

[22] Kumar L., Shamsuzzama, Jadiya P., Haque R., Shukla S., Nazir A. (2018). Functional Characterization of Novel Circular RNA Molecule, circzip-2 and Its Synthesizing Gene zip-2 in *C. elegans* Model of Parkinson’s Disease. Mol. Neurobiol..

[23] Wang M., Yu F., Wu W., Zhang Y., Chang W., Ponnusamy M., Wang K., Li P. (2017). Circular RNAs: A novel type of non-coding RNA and their potential implications in antiviral immunity. Int. J. Biol. Sci..

[24] Medina J.M., Abbas M.N., Bensaoud C., Hackenberg M., Kotsyfakis M. (2022). Bioinformatic Analysis of Ixodes ricinus Long Non-Coding RNAs Predicts Their Binding Ability of Host miRNAs. Int. J. Mol. Sci..

[25] Arnberg A.C., Van Ommen G.J., Grivell L.A., Van Bruggen E.F., Borst P. (1980). Some yeast mitochondrial RNAs are circular. Cell.

[26] Chen L.L., Yang L. (2015). Regulation of circRNA biogenesis. RNA Biol..

[27] Holdt L.M., Kohlmaier A., Teupser D. (2018). Molecular roles and function of circular RNAs in eukaryotic cells. Cell. Mol. Life Sci. CMLS.

[28] Chen I., Chen C.Y., Chuang T.J. (2015). Biogenesis, identification, and function of exonic circular RNAs. Wiley Interdiscip. Rev. RNA.

[29] Zhang Y., Zhang X.O., Chen T., Xiang J.F., Yin Q.F., Xing Y.H., Zhu S., Yang L., Chen L.L. (2013). Circular intronic long noncoding RNAs. Mol. Cell.

[30] Li Z., Huang C., Bao C., Chen L., Lin M., Wang X., Zhong G., Yu B., Hu W., Dai L. (2015). Exon-intron circular RNAs regulate transcription in the nucleus. Nat. Struct. Mol. Biol..

[31] Starke S., Jost I., Rossbach O., Schneider T., Schreiner S., Hung L.H., Bindereif A. (2015). Exon circularization requires canonical splice signals. Cell Rep..

[32] Liang D., Tatomer D.C., Luo Z., Wu H., Yang L., Chen L.L., Cherry S., Wilusz J.E. (2017). The Output of Protein-Coding Genes Shifts to Circular RNAs When the Pre-mRNA Processing Machinery Is Limiting. Mol. Cell.

[33] Kramer M.C., Liang D., Tatomer D.C., Gold B., March Z.M., Cherry S., Wilusz J.E. (2015). Combinatorial control of Drosophila circular RNA expression by intronic repeats, hnRNPs, and SR proteins. Genes Dev..

[34] Zhou Z., Sun B., Huang S., Zhao L. (2019). Roles of circular RNAs in immune regulation and autoimmune diseases. Cell Death Dis..

[35] Liang D., Wilusz J.E. (2014). Short intronic repeat sequences facilitate circular RNA production. Genes Dev..

[36] Zhang X.O., Wang H.B., Zhang Y., Lu X., Chen L.L., Yang L. (2014). Complementary sequence-mediated exon circularization. Cell.

[37] Ye C.Y., Zhang X., Chu Q., Liu C., Yu Y., Jiang W., Zhu Q.H., Fan L., Guo L. (2017). Full-length sequence assembly reveals circular RNAs with diverse non-GT/AG splicing signals in rice. RNA Biol..

[38] Zaphiropoulos P.G. (1996). Circular RNAs from transcripts of the rat cytochrome P450 2C24 gene: Correlation with exon skipping. Proc. Natl. Acad. Sci. USA.

[39] Barrett S.P., Wang P.L., Salzman J. (2015). Circular RNA biogenesis can proceed through an exon-containing lariat precursor. eLife.

[40] Ashwal-Fluss R., Meyer M., Pamudurti N.R., Ivanov A., Bartok O., Hanan M., Evantal N., Memczak S., Rajewsky N., Kadener S. (2014). circRNA biogenesis competes with pre-mRNA splicing. Mol. Cell.

[41] Li X., Liu C.X., Xue W., Zhang Y., Jiang S., Yin Q.F., Wei J., Yao R.W., Yang L., Chen L.L. (2017). Coordinated circRNA Biogenesis and Function with NF90/NF110 in Viral Infection. Mol. Cell.

[42] Conn S.J., Pillman K.A., Toubia J., Conn V.M., Salmanidis M., Phillips C.A., Roslan S., Schreiber A.W., Gregory P.A., Goodall G.J. (2015). The RNA binding protein quaking regulates formation of circRNAs. Cell.

[43] Dong R., Zhang X.O., Zhang Y., Ma X.K., Chen L.L., Yang L. (2016). CircRNA-derived pseudogenes. Cell Res..

[44] Chen L.L. (2020). The expanding regulatory mechanisms and cellular functions of circular RNAs. Nat. Rev. Mol. Cell Biol..

[45] Hansen T.B., Jensen T.I., Clausen B.H., Bramsen J.B., Finsen B., Damgaard C.K., Kjems J. (2013). Natural RNA circles function as efficient microRNA sponges. Nature.

[46] Memczak S., Jens M., Elefsinioti A., Torti F., Krueger J., Rybak A., Maier L., Mackowiak S.D., Gregersen L.H., Munschauer M. (2013). Circular RNAs are a large class of animal RNAs with regulatory potency. Nature.

[47] Conn V.M., Hugouvieux V., Nayak A., Conos S.A., Capovilla G., Cildir G., Jourdain A., Tergaonkar V., Schmid M. (2017). A circRNA from *SEPALLATA3* regulates splicing of its cognate mRNA through R-loop formation. Nat. Plants.

[48] Guarnerio J., Zhang Y., Cheloni G., Panella R., Mae Katon J., Simpson M., Matsumoto A., Papa A. (2019). Intragenic antagonistic roles of protein and circRNA in tumorigenesis. Cell Res..

[49] Liu Y., Su H., Zhang J., Liu Y., Feng C., Han F. (2020). Back-spliced RNA from retrotransposon binds to centromere and regulates centromeric chromatin loops in maize. PLoS Biol..

[50] Poliseno L., Salmena L., Zhang J., Carver B., Haveman W.J., Pandolfi P.P. (2010). A coding-independent function of gene and pseudogene mRNAs regulates tumour biology. Nature.

[51] Salmena L., Poliseno L., Tay Y., Kats L., Pandolfi P.P. (2011). A ceRNA hypothesis: The Rosetta Stone of a hidden RNA language?. Cell.

[52] Bosson A.D., Zamudio J.R., Sharp P.A. (2014). Endogenous miRNA and target concentrations determine susceptibility to potential ceRNA competition. Mol. Cell.

[53] Denzler R., Agarwal V., Stefano J., Bartel D.P., Stoffel M. (2014). Assessing the ceRNA hypothesis with quantitative measurements of miRNA and target abundance. Mol. Cell.

[54] Huang R., Zhang Y., Han B., Bai Y., Zhou R., Gan G., Chao J., Hu G., Yao H. (2017). Circular RNA HIPK2 regulates astrocyte activation via cooperation of autophagy and ER stress by targeting MIR124-2HG. Autophagy.

[55] Zheng Q., Bao C., Guo W., Li S., Chen J., Chen B., Luo Y., Lyu D., Li Y., Shi G. (2016). Circular RNA profiling reveals an abundant circHIPK3 that regulates cell growth by sponging multiple miRNAs. Nat. Commun..

[56] Stoll L., Sobel J., Rodriguez-Trejo A., Guay C., Lee K., Venø M.T., Kjems J., Laybutt D.R., Regazzi R. (2018). Circular RNAs as novel regulators of β-cell functions in normal and disease conditions. Mol. Metab..

[57] Du W.W., Yang W., Liu E., Yang Z., Dhaliwal P., Yang B.B. (2016). Foxo3 circular RNA retards cell cycle progression via forming ternary complexes with p21 and CDK2. Nucleic Acids Res..

[58] Du W.W., Yang W., Chen Y., Wu Z.K., Foster F.S., Yang Z., Li X., Yang B.B. (2017). Foxo3 circular RNA promotes cardiac senescence by modulating multiple factors associated with stress and senescence responses. Eur. Heart J..

[59] Li Q., Wang Y., Wu S., Zhou Z., Ding X., Shi R., Thorne R.F., Zhang X.D., Hu W., Wu M. (2019). CircACC1 Regulates Assembly and Activation of AMPK Complex under Metabolic Stress. Cell Metab..

[60] Burd C.E., Jeck W.R., Liu Y., Sanoff H.K., Wang Z., Sharpless N.E. (2010). Expression of linear and novel circular forms of an INK4/ARF-associated non-coding RNA correlates with atherosclerosis risk. PLoS Genet..

[61] Holdt L.M., Stahringer A., Sass K., Pichler G., Kulak N.A., Wilfert W., Kohlmaier A., Herbst A., Northoff B.H., Nicolaou A. (2016). Circular non-coding RNA ANRIL modulates ribosomal RNA maturation and atherosclerosis in humans. Nat. Commun..

[62] Abdelmohsen K., Panda A.C., Munk R., Grammatikakis I., Dudekula D.B., De S., Kim J., Noh J.H., Kim K.M., Martindale J.L. (2017). Identification of HuR target circular RNAs uncovers suppression of PABPN1 translation by CircPABPN1. RNA Biol..

[63] Liu Q., Kausar S., Tang Y., Huang W., Tang B., Abbas M.N., Dai L. (2022). The Emerging Role of STING in Insect Innate Immune Responses and Pathogen Evasion Strategies. Front. Immunol..

[64] Chen X., Shi W., Chen C. (2019). Differential circular RNAs expression in ovary during oviposition in honey bees. Genomics.

[65] Chen X., Wang D., An J. (2023). Circular RNA ame_circ_2015 Function as microRNA Sponges in Regulating Egg-Laying of Honeybees (*Apis mellifera*). Life.

[66] Zhang Q., Dou W., Pan D., Chen E.H., Niu J.Z., Smagghe G., Wang J.J. (2019). Genome-Wide Analysis of MicroRNAs in Relation to Pupariation in Oriental Fruit Fly. Front. Physiol..

[67] Zhang J., Wen D., Li E.Y., Palli S.R., Li S., Wang J., Liu S. (2021). MicroRNA miR-8 promotes cell growth of corpus allatum and juvenile hormone biosynthesis independent of insulin/IGF signaling in Drosophila melanogaster. Insect Biochem. Mol. Biol..

[68] Hall H., Medina P., Cooper D.A., Escobedo S.E., Rounds J., Brennan K.J., Vincent C., Miura P., Doerge R., Weake V.M. (2017). Transcriptome profiling of aging Drosophila photoreceptors reveals gene expression trends that correlate with visual senescence. BMC Genom..

[69] Weigelt C.M., Sehgal R., Tain L.S., Cheng J., Eßer J., Pahl A., Dieterich C., Grönke S., Partridge L. (2020). An Insulin-Sensitive Circular RNA that Regulates Lifespan in Drosophila. Mol. Cell.

[70] Sun Z., Lu Y., Zhang H., Kumar D., Liu B., Gong Y., Zhu M., Zhu L., Liang Z., Kuang S. (2016). Effects of BmCPV Infection on Silkworm Bombyx mori Intestinal Bacteria. PLoS ONE.

[71] Ito K., Ponnuvel K.M. (2021). Host Response against Virus Infection in an Insect: Bidensovirus Infection Effect on Silkworm (*Bombyx mori*). Antioxidants.

[72] Li H., Li K., Lai W., Li X., Wang H., Yang J., Chu S., Wang H., Kang C., Qiu Y. (2018). Comprehensive circular RNA profiles in plasma reveals that circular RNAs can be used as novel biomarkers for systemic lupus erythematosus. Clin. Chim. Acta Int. J. Clin. Chem..

[73] Abbas M.N., Liang H., Kausar S., Dong Z., Cui H. (2020). Zinc finger protein RP-8, the Bombyx mori ortholog of programmed cell death 2, regulates cell proliferation. Dev. Comp. Immunol..

[74] Kausar S., Abbas M.N., Cui H. (2021). A review on the DNA methyltransferase family of insects: Aspect and prospects. Int. J. Biol. Macromol..

[75] Kausar S., Gul I., Liu R., Ke X.X., Dong Z., Abbas M.N., Cui H. (2022). *Antheraea pernyi* Suppressor of Cytokine Signaling 2 Negatively Modulates the JAK/STAT Pathway to Attenuate Microbial Infection. Int. J. Mol. Sci..

[76] Liu W., Liang W., Xiong X.P. (2022). A circular RNA *Edis*-Relish-*castor* axis regulates neuronal development in *Drosophila*. PLoS Genet..

[77] Zhang K., Su J., Hu X., Yan X., Chen S., Li C., Pan G., Chang H., Tian W., Abbas M.N. (2022). Integrin β2 and β3: Two plasmatocyte markers deepen our understanding of the development of plasmatocytes in the silkworm *Bombyx mori*. Insect Sci..

[78] Hu X., Zhang B., Zheng X., Ji H., Feng K., Hu X., Gul I., Abbas M.N., Cui H., Zhu Y. (2022). Molecular Characterization of Two Genes Encoding Novel Ca^2+^-Independent Phospholipase A2s from the Silkworm, *Bombyx mori*. Curr. Issues Mol. Biol..

[79] Abbas M.N., Kausar S., Gul I., Ke X.X., Dong Z., Lu X., Cui H. (2021). Suppressor of cytokine signalling 6 is a potential regulator of antimicrobial peptides in the Chinese oak silkworm, *Antheraea pernyi*. Mol. Immunol..

[80] Chen H., Fan X., Zhang W., Ye Y., Cai Z., Zhang K., Zhang K., Fu Z., Chen D., Guo R. (2022). Deciphering the CircRNA-Regulated Response of Western Honey Bee (*Apis mellifera*) Workers to Microsporidian Invasion. Biology.

[81] Hu X., Zhu M., Zhang X., Liu B., Liang Z., Huang L., Xu J., Yu L., Li K., Zar M.S. (2018). Identification and characterization of circular RNAs in the silkworm midgut following *Bombyx mori* cytoplasmic polyhedrosis virus infection. RNA Biol..

[82] Wu P., Qin G., Qian H., Chen T., Guo X. (2016). Roles of miR-278-3p in IBP2 regulation and *Bombyx mori* cytoplasmic polyhedrosis virus replication. Gene.

[83] Abbas M.N., Kausar S., Zhao E., Cui H. (2020). Suppressors of cytokine signaling proteins as modulators of development and innate immunity of insects. Dev. Comp. Immunol..

[84] Zhang S., Shen M. (2020). Expression profile analysis of circular RNAs in BmN cells (*Bombyx mori*) upon BmNPV infection. Arch. Insect Biochem. Physiol..

[85] Feng M., Kolliopoulou A., Zhou Y.H., Fei S.G., Xia J.M., Swevers L., Sun J.C. (2021). The piRNA response to BmNPV infection in the silkworm fat body and midgut. Insect Sci..

[86] Wang L., Xiao Q., Zhou X.L., Zhu Y., Dong Z.Q., Chen P., Pan M.H., Lu C. (2017). *Bombyx mori* Nuclear Polyhedrosis Virus (BmNPV) Induces Host Cell Autophagy to Benefit Infection. Viruses.

[87] Yin H., Zhang S., Shen M., Zhang Z., Huang H., Zhao Z., Guo X., Wu P. (2021). Integrative analysis of circRNA/miRNA/mRNA regulatory network reveals the potential immune function of circRNAs in the *Bombyx mori* fat body. J. Invertebr. Pathol..

[88] Hu X., Zhu M., Liu B., Liang Z., Huang L., Xu J., Yu L., Li K., Jiang M., Xue R. (2018). Circular RNA alterations in the Bombyx mori midgut following *B. mori* nucleopolyhedrovirus infection. Mol. Immunol..

[89] Zhang J., Wang H., Wu W., Dong Y., Wang M., Yi D., Zhou Y., Xu Q. (2020). Systematic Identification and Functional Analysis of Circular RNAs During Rice Black-Streaked Dwarf Virus Infection in the *Laodelphax striatellus* (Fallén) Midgut. Front. Microbiol..

[90] Somu C., Karuppiah H., Sundaram J. (2019). Antiviral activity of seselin from Aegle marmelos against nuclear polyhedrosis virus infection in the larvae of silkworm, *Bombyx mori*. J. Ethnopharmacol..

[91] Wu P., Shang Q., Dweteh O.A., Huang H., Zhang S., Zhong J., Hou Q., Guo X. (2019). Over expression of bmo-miR-2819 suppresses BmNPV replication by regulating the BmNPV ie-1 gene in *Bombyx mori*. Mol. Immunol..

[92] Yu H., Wang X., Xu J., Ma Y., Zhang S., Yu D., Fei D., Muhammad A. (2017). iTRAQ-based quantitative proteomics analysis of molecular mechanisms associated with *Bombyx mori* (Lepidoptera) larval midgut response to BmNPV in susceptible and near-isogenic strains. J. Proteom..

[93] Deddouche S., Matt N., Budd A., Mueller S., Kemp C., Galiana-Arnoux D., Dostert C., Antoniewski C., Hoffmann J.A., Imler J.L. (2008). The DExD/H-box helicase Dicer-2 mediates the induction of antiviral activity in drosophila. Nat. Immunol..

[94] Paradkar P.N., Trinidad L., Voysey R., Duchemin J.B., Walker P.J. (2012). Secreted Vago restricts West Nile virus infection in Culex mosquito cells by activating the Jak-STAT pathway. Proc. Natl. Acad. Sci. USA.

[95] Asad S., Parry R., Asgari S. (2018). Upregulation of Aedes aegypti Vago1 by Wolbachia and its effect on dengue virus replication. Insect Biochem. Mol. Biol..

[96] Sun L., Liu S., Chen Z.J. (2010). SnapShot: Pathways of antiviral innate immunity. Cell.

[97] Goubau D., Deddouche S., Reis e Sousa C. (2013). Cytosolic sensing of viruses. Immunity.

[98] Roers A., Hiller B., Hornung V. (2016). Recognition of Endogenous Nucleic Acids by the Innate Immune System. Immunity.

[99] Cadena C., Hur S. (2017). Antiviral Immunity and Circular RNA: No End in Sight. Mol. Cell.

[100] Chen Y.G., Kim M.V., Chen X., Batista P.J., Aoyama S., Wilusz J.E., Iwasaki A., Chang H.Y. (2017). Sensing Self and Foreign Circular RNAs by Intron Identity. Mol. Cell.

[101] Chu S.H., Liu L., Abbas M.N., Li Y.Y., Kausar S., Qian X.Y., Ye Z.Z., Yu X.M., Li X.K., Liu M. (2019). Peroxiredoxin 6 modulates Toll signaling pathway and protects DNA damage against oxidative stress in red swamp crayfish (*Procambarus clarkii*). Fish Shellfish Immunol..

[102] Dai L.S., Kausar S., Gul I., Zhou H.L., Abbas M.N., Deng M.J. (2020). Molecular characterization of a heat shock protein 21 (Hsp21) from red swamp crayfish, *Procambarus clarkii* in response to immune stimulation. Dev. Comp. Immunol..

[103] Zhang K., Shen L., Wang X., Yang H., Zhang X., Pan G., Li C., Ji H., Abbas M.N., Li C. (2021). Scavenger receptor C regulates antimicrobial peptide expression by activating toll signaling in silkworm, *Bombyx mori*. Int. J. Biol. Macromol..

[104] Xiong X.P., Liang W., Liu W. (2022). The circular RNA Edis regulates neurodevelopment and innate immunity. PLoS Genet..

[105] Rybak-Wolf A., Stottmeister C., Glažar P., Jens M., Pino N., Giusti S., Hanan M., Behm M., Bartok O., Ashwal-Fluss R. (2015). Circular RNAs in the Mammalian Brain Are Highly Abundant, Conserved, and Dynamically Expressed. Mol. Cell.

[106] Akhouayri I., Turc C., Royet J., Charroux B. (2011). Toll-8/Tollo negatively regulates antimicrobial response in the Drosophila respiratory epithelium. PLoS Pathog..

[107] Legnini I., Di Timoteo G., Rossi F., Morlando M., Briganti F., Sthandier O., Fatica A., Santini T., Andronache A., Wade M. (2017). Circ-ZNF609 Is a Circular RNA that Can Be Translated and Functions in Myogenesis. Mol. Cell.

[108] Pamudurti N.R., Bartok O., Jens M., Ashwal-Fluss R., Stottmeister C., Ruhe L., Hanan M., Wyler E., Perez-Hernandez D., Ramberger E. (2017). Translation of CircRNAs. Mol. Cell.

[109] Shang Y., Feng P., Wang C. (2015). Fungi That Infect Insects: Altering Host Behavior and Beyond. PLoS Pathog..

[110] Sun Y.X., Zhu B.J., Tang L., Sun Y., Chen C., Nadeem Abbas M., Wang L., Qian C., Wei G.Q., Liu C.L. (2017). Cathepsin O is involved in the innate immune response and metamorphosis of Antheraea pernyi. J. Invertebr. Pathol..

[111] Sun Y.X., Tang L., Wang P., Abbas M.N., Tian J.W., Zhu B.J., Liu C.L. (2018). Cathepsin L-like protease can regulate the process of metamorphosis and fat body dissociation in *Antheraea pernyi*. Dev. Comp. Immunol..

[112] Abbas M.N., Kausar S., Sun Y.X., Sun Y., Wang L., Qian C., Wei G.Q., Zhu B.J., Liu C.L. (2017). Molecular cloning, expression, and characterization of E2F transcription factor 4 from *Antheraea pernyi*. Bull. Entomol. Res..

[113] Ke X.X., Chao H., Abbas M.N., Kausar S., Gul I., Ji H., Yang L., Cui H. (2020). Niemann-Pick type C1 regulates cholesterol transport and metamorphosis in silkworm, *Bombyx mori* (Dazao). Int. J. Biol. Macromol..

[114] Genersch E. (2010). Honey bee pathology: Current threats to honey bees and beekeeping. Appl. Microbiol. Biotechnol..

[115] Ye Y., Fan X., Cai Z., Wu Y., Zhang W., Zhao H., Guo S., Feng P., Li Q., Zou P. (2022). Unveiling the circRNA-Mediated Immune Responses of Western Honey Bee Larvae to *Ascosphaera apis* Invasion. Int. J. Mol. Sci..

[116] Vieira J., Freitas F.C.P. (2021). miRNA-34 and miRNA-210 target hexamerin genes enhancing their differential expression during early brain development of honeybee (*Apis mellifera*) castes. Insect Mol. Biol..

[117] Zhu Z., Wang J., Fan X., Long Q., Chen H., Ye Y., Zhang K., Ren Z., Zhang Y., Niu Q. (2022). CircRNA-regulated immune responses of asian honey bee workers to microsporidian infection. Front. Genet..

[118] Han D., Wang Y., Wang Y., Dai X., Zhou T., Chen J., Tao B., Zhang J., Cao F. (2020). The Tumor-Suppressive Human Circular RNA CircITCH Sponges miR-330-5p to Ameliorate Doxorubicin-Induced Cardiotoxicity Through Upregulating SIRT6, Survivin, and SERCA2a. Circ. Res..

[119] Li J., Huang C., Zou Y., Ye J., Yu J., Gui Y. (2020). CircTLK1 promotes the proliferation and metastasis of renal cell carcinoma by sponging miR-136-5p. Mol. Cancer.

[120] Zhao J., Lee E.E., Kim J. (2019). Transforming activity of an oncoprotein-encoding circular RNA from human papillomavirus. Nat. Commun..

[121] Zhu M., Liang Z., Pan J., Zhang X., Xue R., Cao G., Hu X., Gong C. (2021). Hepatocellular carcinoma progression mediated by hepatitis B virus-encoded circRNA HBV_circ_1 through interaction with CDK1. Mol. Ther. Nucleic Acids.

[122] Zhang Y., Zhang X., Dai K., Zhu M., Liang Z., Pan J., Zhang Z., Xue R., Cao G., Hu X. (2022). Bombyx mori Akirin hijacks a viral peptide vSP27 encoded by BmCPV circRNA and activates the ROS-NF-κB pathway against viral infection. Int. J. Biol. Macromol..

[123] Zhang Y., Zhu M., Zhang X., Dai K., Liang Z., Pan J., Zhang Z., Cao M., Xue R., Cao G. (2022). Micropeptide vsp21 translated by Reovirus circular RNA 000048 attenuates viral replication. Int. J. Biol. Macromol..

[124] Zhang X., Liang Z., Wang C., Shen Z., Sun S., Gong C., Hu X. (2022). Viral Circular RNAs and Their Possible Roles in Virus-Host Interaction. Front. Immunol..

[125] Hikida H., Kokusho R., Matsuda-Imai N., Katsuma S. (2020). Bombyx mori nucleopolyhedrovirus Bm96 suppresses viral virulence in *Bombyx mori* larvae. J. Invertebr. Pathol..

[126] Zhang Y., Zhang X., Shen Z., Qiu Q., Tong X., Pan J., Zhu M., Hu X., Gong C. (2023). BmNPV circular RNA-encoded peptide VSP39 promotes viral replication. Int. J. Biol. Macromol..

[127] Fan X., Yang Y., Chen C. (2022). Pervasive translation of circular RNAs driven by short IRES-like elements. Nat. Commun..

[128] Shi Y., Jia X. (2020). The new function of circRNA: Translation. Clin. Transl. Oncol..

[129] Guo R., Chen D., Chen H., Fu Z., Xiong C., Hou C., Zheng Y., Guo Y., Wang H., Du Y. (2018). Systematic investigation of circular RNAs in *Ascosphaera apis*, a fungal pathogen of honeybee larvae. Gene.

[130] Hu X., Chen F., Zhu L., Yu L., Zhu M., Liang Z., Zhang X., Xue R., Cao G., Gong C. (2019). *Bombyx mori* cypovirus encoded small peptide inhibits viral multiplication. Dev. Comp. Immunol..

[131] Chen C., Yang L., Abbas M.N., Zou D., Li J., Geng X., Zhang H., Sun Y. (2022). Relish regulates innate immunity via mediating ATG5 activity in *Antheraea pernyi*. Dev. Comp. Immunol..

[132] Hua X., Li B., Song L., Hu C., Li X., Wang D., Xiong Y., Zhao P., He H., Xia Q. (2018). Stimulator of interferon genes (STING) provides insect antiviral immunity by promoting Dredd caspase-mediated NF-κB activation. J. Biol. Chem..

[133] O’Reilly D.R., Miller L.K. (1991). Improvement of a Baculovirus Pesticide by Deletion of the *EGT* Gene. Bio/Technol..

[134] Katsuma S., Shimada T. (2015). The killing speed of egt-inactivated *Bombyx mori* nucleopolyhedrovirus depends on the developmental stage of *B. mori* larvae. J. Invertebr. Pathol..

